# Case report: preoperative radiological diagnosis of uterine torsion in the non-gravid uterus

**DOI:** 10.1093/bjrcr/uaaf010

**Published:** 2025-04-04

**Authors:** Senthan Rudrakumar, Kaivalya Bhagat, Lauren Jane Matthews, Ashish Bhagat, Vivek Malhotra

**Affiliations:** West Herts NHS Trust, Hertfordshire, WD18 0HB, United Kingdom; Luton and Dunstable NHS Trust, Bedfordshire, LU4 0DZ, United Kingdom; West Herts NHS Trust, Hertfordshire, WD18 0HB, United Kingdom; West Herts NHS Trust, Hertfordshire, WD18 0HB, United Kingdom; West Herts NHS Trust, Hertfordshire, WD18 0HB, United Kingdom

**Keywords:** uterine, fibroid, torsion, uterine torsion, infarction, leiomyoma, MRI, CT, small bowel obstruction, pelvis mass

## Abstract

This case report describes the rare occurrence of a pre-operative radiological diagnosis of uterine torsion in the non-gravid uterus. A 78-year-old female presented with a 5-day history of worsening non-specific gastrointestinal symptoms. Her admission CT study initially reported a large adnexal mass lesion causing obstruction of neighbouring small bowel loops. Management was initiated under the presumption of a complex uterine fibroid causing local small bowel obstruction. Only a secondary radiological review—conducted due to persistent abdominal pain—identified the characteristic “whirlpool” sign of the uterine cavity and prompted the differential of uterine torsion. Contrast-enhanced MRI study further confirmed this suggestion with a lack of uterine contrast uptake and the “X-sign.” The patient subsequently had an emergency laparotomy for a total abdominal hysterectomy and bilateral salpingo-oophorectomy. Intra-operative findings and further histological analysis demonstrated a distorted uterine cavity with haemorrhagic infarction, confirming a diagnosis of uterine torsion.

## Case presentation

A 78-year-old woman who initially presented to her family doctor and was subsequently transferred to the local A&E department reported a 5-day history of right lower quadrant pain, bloating, and nausea with associated worsening constipation. On initial assessment, the patient was tachycardic and febrile. She did not have any prior surgical or medical history of note.

## Admission/investigations

**Figure 1.**
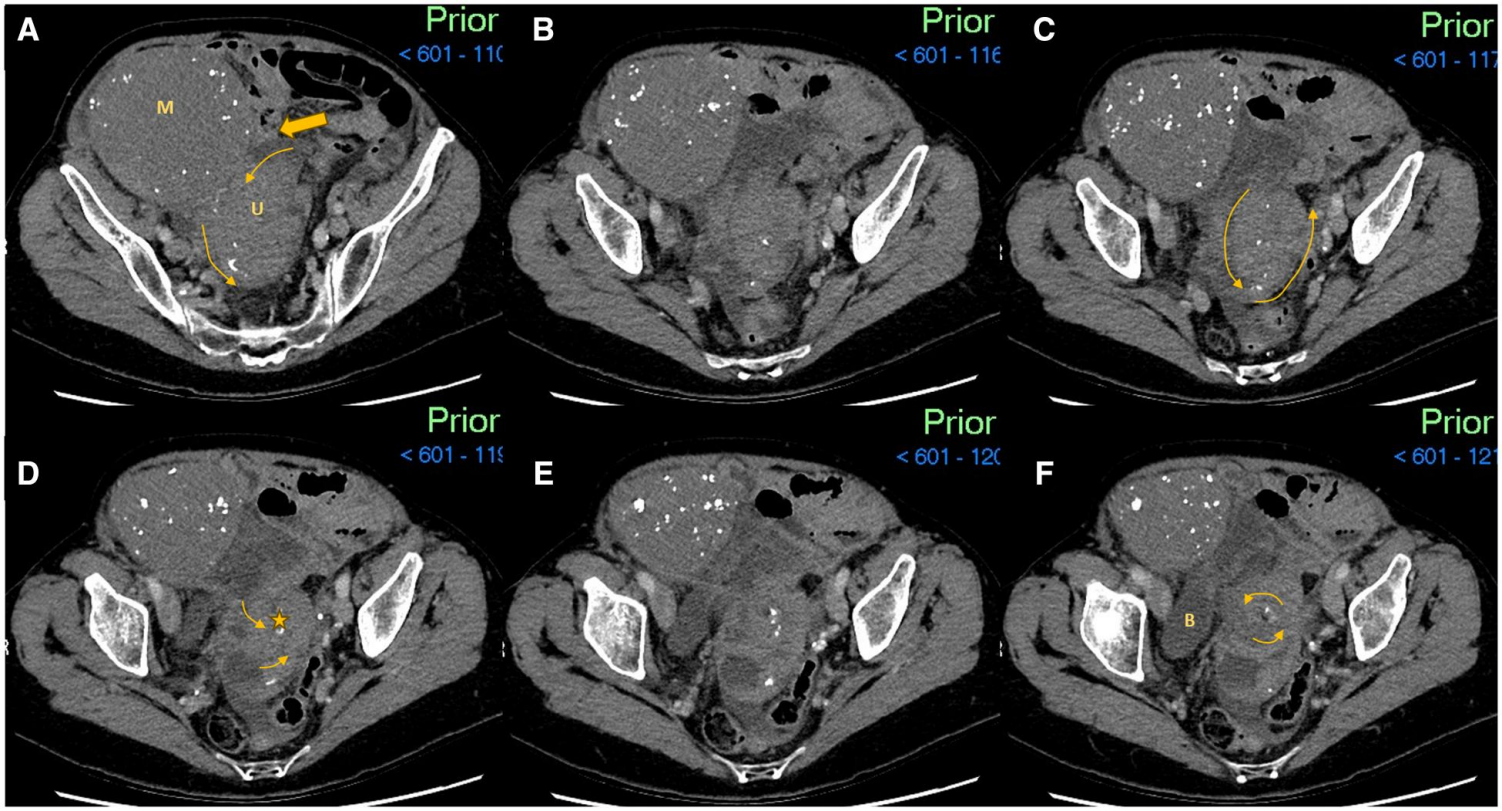
Admission CT abdomen and pelvis with contrast with selected axial images in the abdominal window setting (A-F caudally). (A) A large 17 × 11.5 × 11.3 cm fibroid mass (M) with peripheral calcifications arising from the uterine fundus. The uterus (U) is seen in the midline pelvis with the commencement of the whirling as represented by the smaller arrow heads. Upstream small bowel dilatation is not fully captured on key images, however, a point of small bowel tethering (block arrow) is noted at the uterine fundal portion. (B-F) Scrolling caudally from slices B to F the U appears to be following an anti-clockwise spiralling appearance “(whirlpool sign)”. This is best visualized on dynamic images however every effort has been made to highlight this on the following static images. The twisting/whirling is occurring around the centre point cervix highlighted by the star in image D. This is then appreciated on E and F as a hypodense centre point focus. The whirling is occurring in the axial plane and therefore sagittal and coronal CT reformats do not show the classic whirling pattern. The curved arrows (A-F) aim to depict the 540-degree rotation of the uterus around the centre point cervix, with the rotation spiralling circumference gradually getting narrower with caudal progression. The uterine wall calcifications are further supportive of the whirling appearance with calcifications being more peripheral in A and B and then more central on E and F. (E) This demonstrates speckles of calcifications entering the “vortex/epicentre,” (cervix). Additionally, the morphology of the calcifications appear comma shaped, which helps to indicate the direction of travel of the calcification and therefore alignment of the underlying uterine tissue. Abbreviations: M = uterine mass, suspected large fibroid; U = Uterus; Block arrow = point of small bowel tethering onto the uterine fundus and mass; Star (D) = cervical canal, centre point of rotation. Image slices A-F (cranial to caudal sequences).

Initial blood tests demonstrated raised inflammatory markers and evidence of neutrophilia, with a C-reactive protein (CRP) of 252 mg/L (normal 0-5 mg/L) and neutrophils of 23 × 10^9^/L (normal 1.5-7.2 × 10^9^/L). The CT abdomen and pelvis (Figure 1) organized on day 1 of presentation demonstrated a large 17 × 11.5 × 11.3 cm mass lesion of possible ovarian aetiology within the right lower quadrant, which was causing partial obstruction of the nearby small bowel loops. The patient was subsequently admitted under the joint care of the surgical and gynaecological team and due to the raised inflammatory markers, she received intravenous antibiotics for a suspected ovarian tumour with an infective component. A nasogastric tube was inserted in light of her obstructive symptoms. On day 5 of admission, the local gynaecological multidisciplinary team (MDT) reached a consensus view that this could represent a sub-serosal (FIGO 6) complex uterine fibroid causing local small bowel obstruction. Due to an improvement of the patient’s symptoms with conservative management alone, a non-emergency myomectomy was planned. However, on day 10 of admission, the patient became febrile and reported further episodic abdominal pain.

Further review of the initial CT images (Figure 1) revealed a “swirling” or “whorled” structure which appeared to spin 540° on its axis, located adjacent to the previously described large mass. These findings raised suspicion of uterine torsion with the mass likely representative of a large uterine fibroid. A contrast-enhanced MRI of the pelvis performed as part of the general work-up for a gynaecological malignancy, further supported the diagnosis of uterine torsion.

## Differential diagnosis

**Figure 2.**
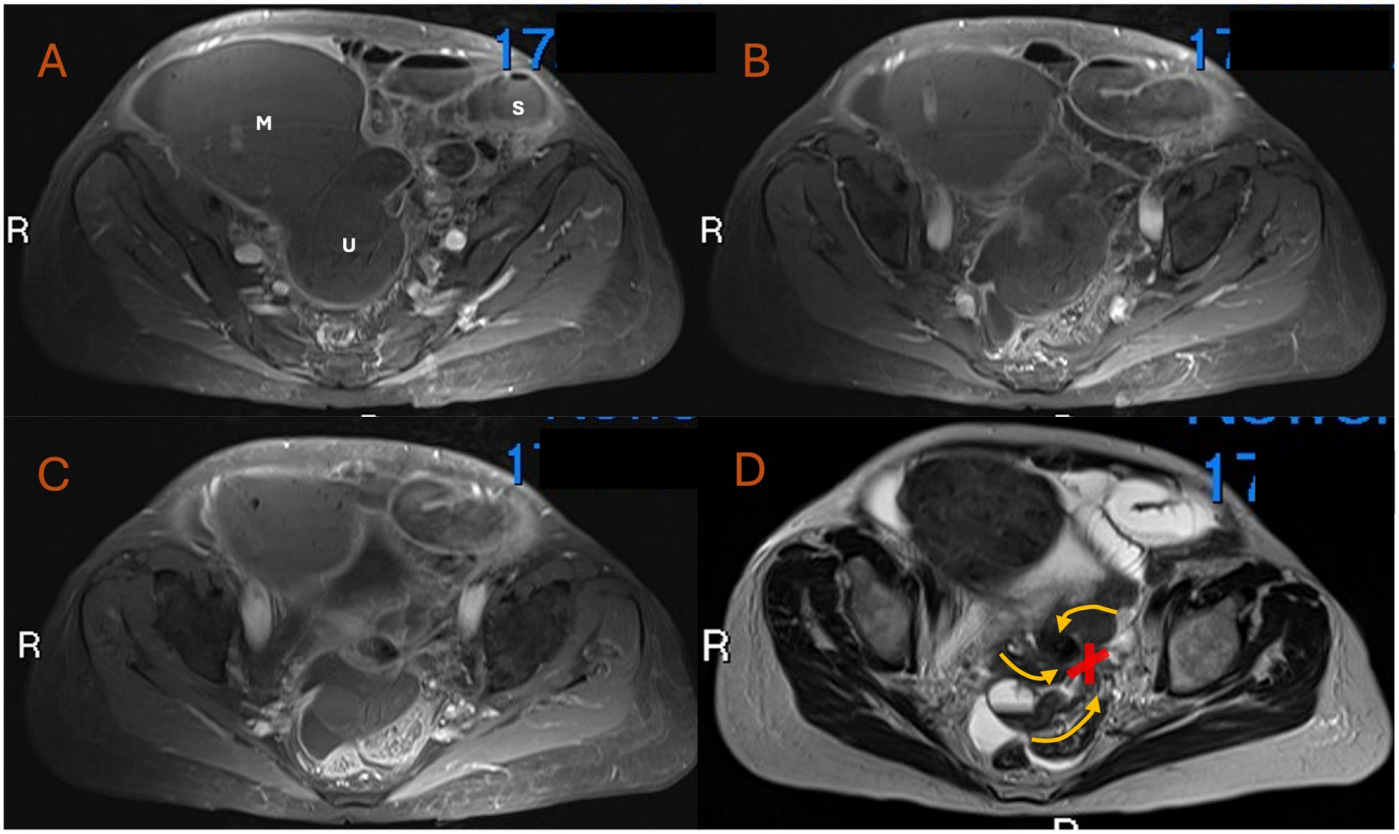
Day 12 of admission MRI pelvis with contrast—axial slices. (A-C) *Post-contrast axial T1-weighted fat suppressed images* moving caudally with the uterine fibroid mass (M) and uterus (U) identified. The key finding is the total lack of contrast uptake within the uterus, adnexal structures and associated fibroid mass highly suggestive of a local ischaemic cause. Ongoing mild small bowel upstream dilatation noted (S). The background “whirlpool sign” is again demonstrated of the un-enhancing uterine cavity. (D) *Annotated non-contrast T2-weighted axial image* of the same slice as C depicts the “X sign” which is a central focal point of tethering to the left of the uterine cervix/vaginal wall where the uterus and adnexal structures appear to wrap around. The wrapping adnexal structures around the “X” are labelled with corresponding curved arrows. The “X-sign” is also demonstrated on the contemporaneous contrast sequence of the same slice (C). Abbreviations: M = Uterine mass, suspected large fibroid; X = “X sign”; S = small bowel; U = Uterus; Curved arrowhead = direction of travel of uterine and adnexal structures.

Prior to the admission CT scan (Figure 1), the differential diagnosis included general acute abdomen differentials such as bowel obstruction, diverticulitis, and appendicitis. Following the visualization of a large, calcified mass on CT study, and in conjunction with the raised inflammatory markers, an ovarian malignancy with a possible infective component was then considered. A subsequent CT thorax was also undertaken for pre-emptive staging purposes. On day 5 of admission, the local MDT suggested additional differential diagnoses including a large degenerative atypical fibroid causing local small bowel obstruction, ovarian torsion, pelvic inflammatory disease, or an appendiceal mass. At day 10 of admission, the fluctuating symptom course prompted a further review of the imaging (Figures 1 and 2) which then raised the possibility of uterine torsion.

## Treatment

Due to the assessment that the patient was biochemically and symptomatically stable, a non-urgent MRI was requested and only reported on day 13 of admission. Following the radiological review of the initial CT and subsequent MRI images (Figures 1 and 2), the possibility of uterine torsion was urgently relayed to the gynaecology team who arranged for the patient to have a mid-line open laparotomy for a total abdominal hysterectomy and bilateral salpingo-oophorectomy. This took place on day 14 of the patient’s admission. The intra-operative findings consisted of a huge avascular and necrotic mass associated with the uterus and ovaries. The small bowel was noted to be tethered over the mass, with surrounding inflammatory adhesions which were surgically released.

## Outcome/follow-up

**Figure 3.**
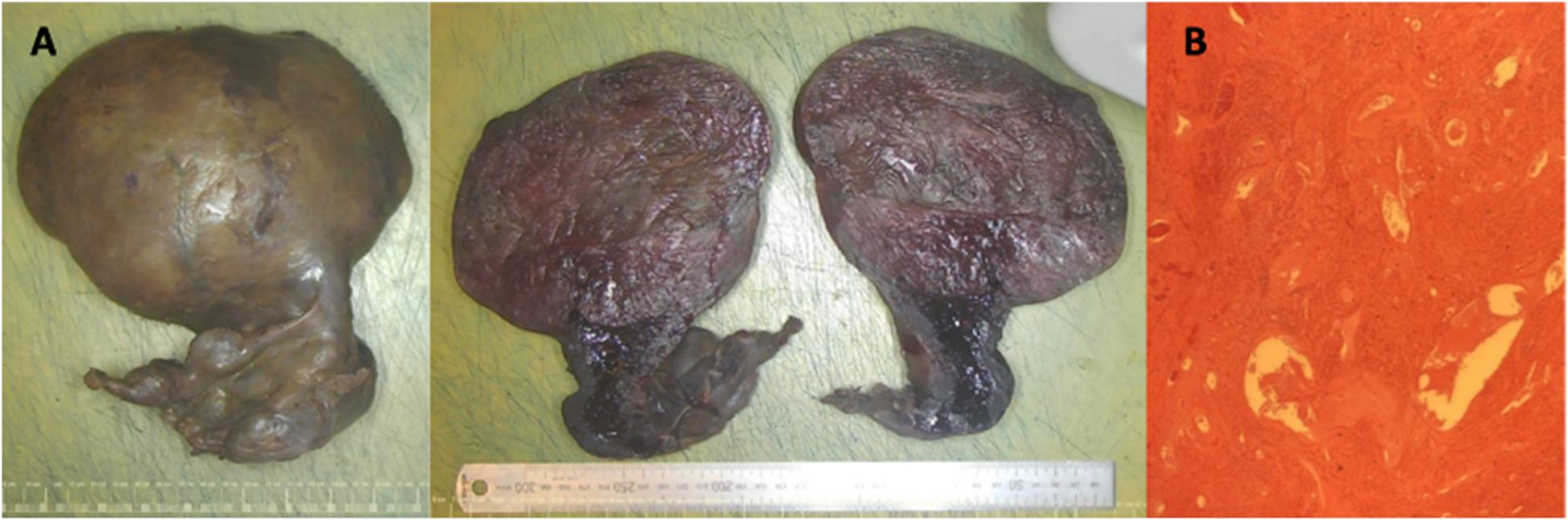
Macroscopic and microscopic images of specimen. (A) Macroscopic photo of the intact specimen shows an enlarged, distorted and haemorrhagic uterus and cervix measuring 180 mm (SI) × 160 mm (ML) × 100 mm (AP). Distortion and adherence of the adnexae to the cervix meant the specimen could not be orientated. Sectioning of the specimen revealed a well-circumscribed mass, measuring 160 × 120 mm, which replaced the entire uterus. The mass was partly calcified and suspected to be a fibroid. (B) Representative histological section from the uterine mass shows features consistent with infarction with widespread haemorrhage, congested blood vessels, and mild inflammation. No viable tissue is seen.

The patient recovered well and was discharged on day 20 of admission, with no major post-operative complications. The specimen was sent to pathology where macroscopic assessment showed global distortion of a grossly enlarged uterus and cervix. Sectioning the specimen revealed a well-circumscribed 160 × 120 mm mass that had taken the place of the uterus (Figure 3). The mass was partly calcified and suspected to be a fibroid. Representative histological sections were taken from the uterine mass, cervix, and adnexae, which showed features consistent with infarction with widespread haemorrhage, congested blood vessels, and mild inflammation. No viable malignancy was identified.

Global distortion of the uterus and cervix, along with histological evidence of widespread haemorrhagic infarction and the aforementioned radiological findings, align with a diagnosis of uterine torsion. The presence of a large FIGO 6 leiomyoma provided a suspected underlying mechanism. The patient was informed of the results with no planned further treatment or ongoing follow-up required.

## Discussion

Uterine torsion is defined as a rotation of >45° around the long axis of the uterus.^1^ Its occurrence is rare, with only 200 cases reported as of 2019.^2^ The majority of cases are associated with pregnancy; between 2001 and 2021, only 26 cases of uterine torsion in non-gravid women were reported, with the commonest cause being uterine leiomyomas, as in this case.^1^ It has been postulated that large sub-serosal fibroids may cause laxity and elongation of the tissues and ligaments forming the lateral attachment of the uterus, therefore causing torsion around the well-anchored cervix.^3^ Asymmetrical enlargement of the uterus may also cause deviation of the axis and therefore rotation.^3^ Unfortunately, its clinical and biochemical presentation is often non-specific and symptoms can range from mild abdominal discomfort to acute pain with symptoms of shock.^1^^,^^4^ There may also be an abdominal mass, pain radiating to the back, nausea and vomiting, gastrointestinal and urinary symptoms, or vaginal bleeding.^4–6^

**Figure 4.**
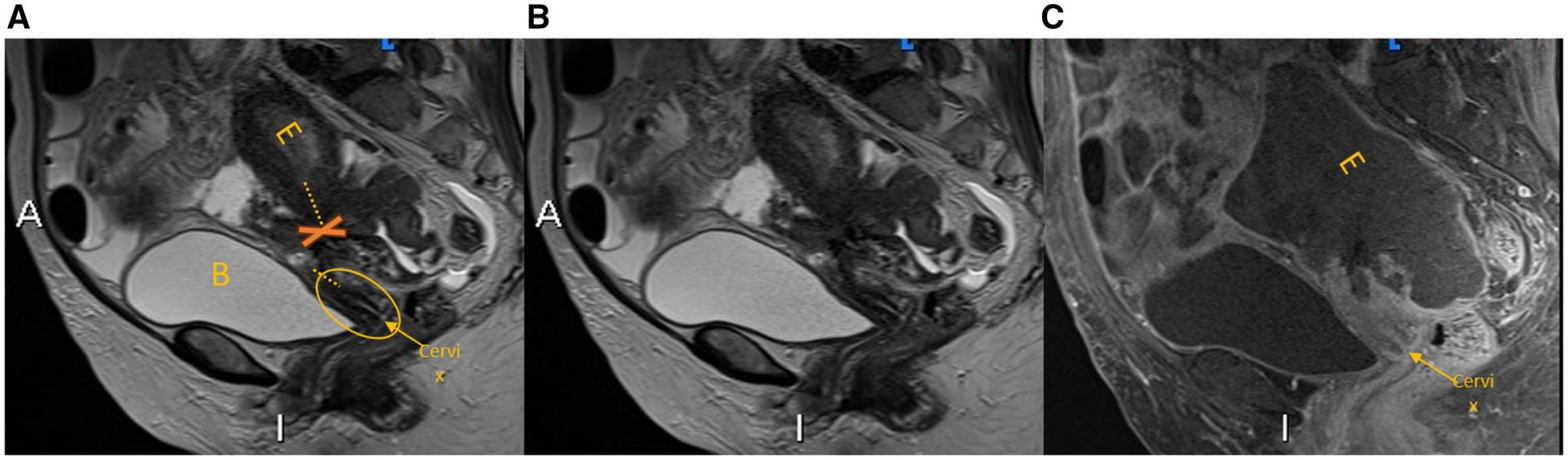
Day 12 of admission MRI pelvis with contrast—sagittal slices. (A and B) *T2-weighted sagittal sequence, both annotated and unannotated*. A (annotated) demonstrates the high T2 signal bladder anteriorly, with the Uterus and T2 bright Endometrial cavity (E) posterior and superior to the bladder. The “X sign” is again appreciable with uterine and adnexal structures tethering between the misaligned uterus and cervix. On A, the cervix and uterine body/fundus (E), do not appear to normally align, with discontinuation of the alignment represented by the dotted lines. (C) *Post-contrast sagittal T1-weighted fat suppressed image at the slice as A and B*: The cervix has again been labelled. The key finding on this image, is the total lack of normally expected uterine and uterine mass enhancement, whereas the misaligned cervix continues to demonstrate enhancement. Abbreviations: B = Bladder; Cervix = labelled; E = Endometrial cavity; X = “X sign”; Dotted line = alignment of uterus and cervix.

Uterine torsion is mostly diagnosed post-operatively with the burden of pre-operative diagnosis being placed on radiology. Pelvic ultrasound can be unremarkable,^3^ and it is the findings on CT scan or MRI that typically help confirm the diagnosis. There may be gas in the uterine cavity, an X-shaped appearance of the upper vagina, malposition of the uterus, or visible ischaemia or infarction of the uterus on contrast-enhanced CT or MRI.^1^^,^^3^^,^^5^^,^^6^ The most characteristic finding on CT or MRI is the “whirl sign” or “whirlpool sign,” as demonstrated in our case occurring in the axial plane, therefore sagittal and coronal reformats were less indicative. The “X sign” has only a few reported instances, however, was appreciated on the MRI sagittal T2 and post-contrast sequences (Figure 4).^1^ The sensitivity of the “whirl sign” which describes a twisted, whorled appearance of the cervix for uterine torsion is 44.4%, with a positive predictive value of 100%.^1^ Of the 26 reported cases of uterine torsion in non-gravid women in the last 20 years however, only 7 (26.9%) were correctly diagnosed pre-operatively.^1^

Uterine torsion is a gynaecological emergency requiring prompt recognition and treatment; if left untreated, it can lead to haemorrhagic infarction and necrosis of the uterus, fallopian tubes, and ovaries.^1^ Complications include intestinal obstruction, haemorrhage, shock, and death. Even once treatment is initiated, there may be long-term impacts on fertility and most young women lose their ability to conceive.^1^^,^^4^ The patient in our case had her emergency surgical treatment following 14 days as an inpatient. The delay in diagnosis was primarily due to a clinical lack of awareness of the diagnosis and a fluctuating symptom course due to partial responsiveness to conservative measures. From a radiological perspective, satisfaction of search, out of hours reporting, and subtlety of the radiological signs were all contributing factors. This uncertainty and delay to diagnosis is also reflected in the literature on uterine torsion.

## Conclusion

Uterine torsion, though rare, is a gynaecological emergency with non-specific clinical features and significant risks if undiagnosed, as seen in this case where delayed recognition led to a complicated clinical course. Early, accurate radiological identification, especially with CT or MRI can significantly improve outcomes and prevent severe complications such as potential loss of fertility.

## Learning points

- Uterine torsion, though rare, should be considered in the differential diagnosis of women presenting with non-specific abdominal pain, particularly in the presence of large uterine fibroids.
- Early diagnosis is crucial; CT and MRI are the most effective imaging modalities, with the “whirl sign” being a key indicator of torsion.
- A high index of suspicion is essential for timely diagnosis, as delays in recognition can lead to severe complications such as shock, bowel obstruction, and fertility loss.
- Open communication and discussion between clinical teams and radiology is key to re-evaluating cases where the clinical picture is discordant to the initial radiological opinion.
